# Treatment-Resistant Immunoproliferative Small Intestinal Disease (IPSID) Leading to Lymphoma

**DOI:** 10.7759/cureus.62302

**Published:** 2024-06-13

**Authors:** Mohamed Ismail, Umair M Nasir, Menna-Allah Elaskandrany, Rajendra Kapila, Weizheng Wang

**Affiliations:** 1 Department of Medicine, Rutgers New Jersey Medical School, Newark, USA; 2 Department of Gastroenterology, NYU Grossman Long Island School of Medicine, Mineola, USA; 3 Department of Medicine, Lenox Hill Hospital, New York, USA; 4 Department of Infectious Diseases, Rutgers New Jersey Medical School, Newark, USA; 5 Department of Gastroenterology and Hepatology, Rutgers New Jersey Medical School, Newark, USA

**Keywords:** mucosa-associated lymphoid tissue (malt), α-heavy chain enteropathy, lymphoma, chronic campylobacter jejuni infection, immunoproliferative small intestinal disease (ipsid)

## Abstract

Immunoproliferative small intestinal disease (IPSID) is a distinct variant of mucosa-associated lymphoid tissue (MALT) lymphoma, often linked to chronic *Campylobacter jejuni* infection. Characterized as an extra-nodal marginal zone B-cell lymphoma, IPSID predominantly affects the proximal small intestine. It features lymphoplasmacytic infiltration and deposition of monotypic α-heavy chains in the lamina propria, leading to blunted intestinal villi, malabsorption, and protein-losing enteropathy. IPSID's clinical spectrum ranges from lymphoid infiltration to malignant diffuse large B-cell lymphoma. Similar to MALT lymphoma, early-stage IPSID can be resolved with antibiotic therapy. This case study documents a 50-year-old Nigerian woman presenting with recurrent watery diarrhea, abdominal pain, and weight loss, unresponsive to antibiotics. A 50-year-old female immigrant from Nigeria presented with recurrent watery diarrhea, abdominal pain, and significant weight loss, all refractory to antibiotic treatment. Initial diagnostic investigations revealed a positive *Campylobacter *stool antigen, mesenteric lymphadenopathy on CT and gallium scans, and diffuse mucosal lymphoplasmacytic infiltration with villi flattening on small bowel biopsies. An octreotide scan identified a reactive mesenteric lymph node, confirmed by surgical biopsy as reactive lymphadenitis. The patient was diagnosed with IPSID and commenced antibiotic therapy, which initially resolved her symptoms. However, she experienced frequent recurrences requiring multiple hospitalizations and repeated courses of intravenous antibiotics. Eventually, the disease progressed to lymphoma, necessitating chemotherapy initiation. This case underscores the diagnostic complexities of IPSID, particularly in distinguishing it from other causes of mesenteric lymphadenopathy. It also highlights the challenges in preventing disease progression from a benign to a malignant state despite appropriate antibiotic treatment. Given IPSID's prevalence in endemic regions, it should be considered in differential diagnoses for similar presentations. Continuous monitoring is crucial to evaluate therapeutic response and mitigate the risk of progression to lymphoma. IPSID presents a significant diagnostic and therapeutic challenge. This case exemplifies the necessity for heightened clinical awareness, especially in patients from endemic regions, and the importance of rigorous monitoring to prevent malignant transformation. Further research is warranted to elucidate the mechanisms behind IPSID progression in certain patients despite repeated antibiotic interventions.

## Introduction

Immunoproliferative small intestinal disease (IPSID), also known as Mediterranean lymphoma or α-heavy chain disease, is a variant form of mucosa-associated lymphoid tissue lymphoma (MALT). IPSID predominantly impacts older children and young adults (ages 10-35, averaging 25-30 years) from low socioeconomic backgrounds in developing regions. It is uncommon in both younger children and older adults. Most cases have been documented in the Middle East, North and South Africa, and the Far East. There have also been sporadic reports from other parts of the world, particularly involving immigrants from the Middle East and North Africa [1]. Males are twice as likely to be affected as females, with primary predisposing factors including poor hygiene, inadequate sanitation, and limited access to clean drinking water [2].

It presents with symptoms such as colicky abdominal pain, persistent diarrhea, malabsorption, weight loss, and failure to thrive. Akin to MALT, IPSID is a type of extra-nodal marginal zone B-cell lymphoma that predominantly occurs in the proximal small intestine. It is characterized by lymphoplasmacytic infiltrates with monotypic α-heavy chain expression, lacking an associated light chain [1]. The α-heavy chain proteins accumulate in the lamina propria, leading to the blunting of intestinal villi, malabsorption, and protein-losing enteropathy [3].

The pathology of IPSID can range from lymphoid infiltration to malignant diffuse large B-cell lymphoma [4]. The disease is typically classified into three stages: stage A (benign), stage B (intermediate), and stage C (malignant). Early-stage IPSID can be completely resolved with antibiotic treatment, similar to MALT, which is often associated with chronic *Helicobacter pylori* infection. IPSID, on the other hand, is linked to chronic *Campylobacter jejuni* infection [3,4]. The distinct geographic distribution of IPSID suggests that environmental factors and genetic predisposition may play significant roles in its pathogenesis [3].

We present the case of a West African immigrant diagnosed with stage C IPSID, which progressed to malignant lymphoma. This case highlights the development of IPSID in a previously healthy West African female, associated with chronic gastrointestinal infection by *C. jejuni*, eventually transforming into malignant lymphoma. IPSID should be considered in patients from developing countries who present with persistent chronic diarrhea, weight loss, and mesenteric lymphadenopathy.

## Case presentation

A 50-year-old woman with no prior medical history, who immigrated from Nigeria four months earlier, presented following a syncopal episode. She reported a two-week history of persistent watery diarrhea (up to 10 bowel movements per day), abdominal cramps, and a 30-pound weight loss over six months. She denied experiencing fevers, chills, bloody stool, melena, or tenesmus. Physical examination revealed an ill-appearing, malnourished woman with a diffusely tender abdomen without peritoneal signs. She was previously hospitalized in Nigeria for syncope and dehydration and treated with ciprofloxacin and metronidazole. However, her diarrhea recurred one-week post-discharge.

On admission, she was afebrile with a blood pressure of 89/53 mmHg, heart rate of 103 bpm, respiratory rate of 20, and a BMI of 19 kg/m². Laboratory tests indicated BUN of 4 mg/dL, creatinine of 0.4 mg/dL, albumin of 2.5 mg/dL, aspartate transaminase (AST)/alanine transaminase (ALT) of 35/45 U/L, lactic acid of 1.5 mEq/L, WBC of 15.0 × 10^9^/L, calcium of 8.3 mg/dL, and potassium of 3.2 mEq/L (Table 1). 

**Table 1:** Laboratory values on admission BUN, blood urea nitrogen; AST, aspartate transaminase; ALT, alanine transaminase

Lab test	Patient values	Reference range
WBC	15 K/µl	4-11 K/µl
BUN	4 mg/dL	6-24 mg/dL
Serum creatinine	0.4 mg/dL	0.74-1.35 mg/dL
Albumin	2.5 g/dL	3.5-5.5 g/dL
ALT	45 U/L	0-41 U/L
AST	35 U/L	0-40 U/L
Lactic acid	1.5 mEq/L	< 2 mEq/L
Calcium	8.3 mg/dL	8.5-10.5 mg/dL
Potassium	3.2 mEq/L	3.5-5.2 mEq/L

**Figure 1:**
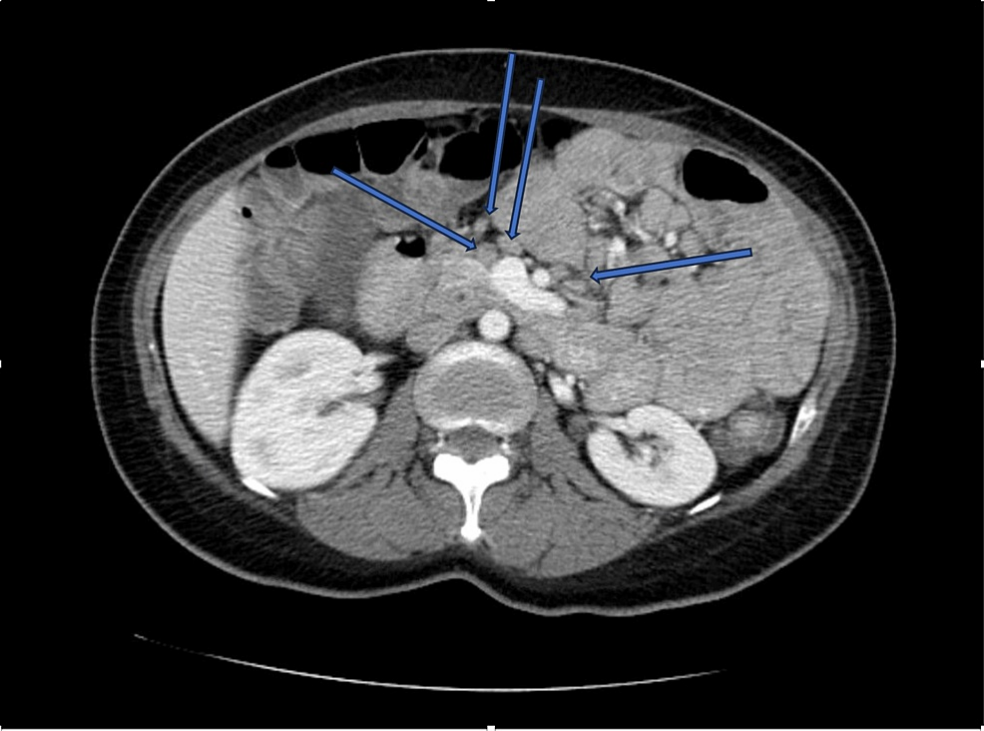
CT of the abdomen and pelvis with intravenous (IV) contrast showing diffuse mesenteric lymphadenopathy

Computed tomography (CT) scan of the abdomen and pelvis with intravenous (IV) contrast revealed diffuse mesenteric lymphadenopathy (Figure 1).

An extensive workup for the etiology of her persistent diarrhea was conducted. Stool analysis was negative for ova and parasites, *Clostridium difficile*, *Cryptosporidium*, *Microsporidia*, *Giardia*, *Yersinia*, and *Vibrio*. Serum gastrin and anti-T transglutaminase (TTG) were within normal limits. *Campylobacter* stool antigen was positive. She received aggressive IV hydration, electrolyte repletion, and treatment with levofloxacin, metronidazole, and rifaximin.

Despite treatment, her gastrointestinal symptoms persisted, leading to endoscopy and colonoscopy, which showed mild to moderate gastritis and colitis without specific findings. Duodenal biopsy revealed blunted villi and lymphocytic infiltration, although anti-TTG antibodies were negative. A gallium scan with single-photon emission computed tomography (SPECT) indicated mildly increased uptake in the mid-abdomen corresponding to the enlarged mesenteric lymph nodes observed on CT. Her diarrhea eventually improved with antibiotics, and she was discharged on a gluten-free diet with instructions to maintain a food journal.

One month later, she returned with recurrent diarrhea, epigastric abdominal pain, and several episodes of non-bloody, non-bilious vomiting. She reported strict adherence to a gluten-free diet, and her food journal showed no correlation between food intake and gastrointestinal symptoms. She was hypotensive with electrolyte imbalances. Another extensive workup, including infectious and non-infectious causes (Table 2), was largely negative except for a positive *Campylobacter* stool antigen.

**Table 2:** Summary of the hospitalization and diagnostic workup findings for a patient, detailing various tests and their results over different time periods BUN, blood urea nitrogen; AST, aspartate transaminase; ALT, alanine transaminase; CT, computed tomography; PCR, polymerase chain reaction; TTG, tissue transglutaminase; SPECT, single-photon emission computed tomography; Ag, antigen; PET-CT, positron emission tomography-computed tomography scan; IPSID, immunoproliferative small intestinal disease

Hospitalization	Study type	Result
Initial	Serum blood work	BUN: 4 mg/dL, creatinine: 0.4 mg/dL, calcium: 8.3 mg/dL, potassium: 3.2 meq/L, albumin: 2.5 g/dL, AST/ALT: 35/45 U/L, WBC: 15 K/µl, lactic acid: 1.5 mEq/L, gastrin: within normal limits, anti-T transglutaminase (TTG): within normal limits
	Imaging	CT abdomen/pelvis: diffuse mesenteric lymphadenopathy
	Stool infectious workup	*Campylobacter* stool Ag: positive, Ova and parasites: negative, GI PCR pathogen panel (*Clostridium difficile*, *Cryptosporidium*, *Microsporidia*, *Giardia*, *Yersinia*, *Vibrio*): negative
	Endoscopy/colonoscopy	Moderate gastritis and colitis with no specific findings
	Duodenal biopsy	Blunted villi and lymphocytic infiltration, anti-TTG immunohistochemistry: negative
	Gallium scan with SPECT	Mild increased uptake within the mid-abdomen corresponding to known mesenteric lymph nodes
One month later	Stool infectious workup	*Campylobacter* stool Ag: positive
	Capsule endoscopy	Edema and hyperemia of small bowel walls Intermittent areas of inflammation
	Small bowel biopsy (Figure 2A, 2B)	Diffuse lymphoplasmacytic infiltration of mucosa with focal lymphoid clusters. Blunting and focal flattening of villi Intraepithelial lymphocytosis and mild crypt hyperplasia. Plasma cell infiltration of the lamina propria
	Octreotide scan	Focus of intense increased uptake in the mesenteric lymph node in the right lower quadrant
	Mesenteric lymph node biopsy	Reactive lymphadenitis
One year later	PET-CT	Cervical and inguinal lymphadenopathy with increased uptake
	Cervical lymph node biopsy	IPSID stage C with transformation to lymphoma

**Figure 2:**
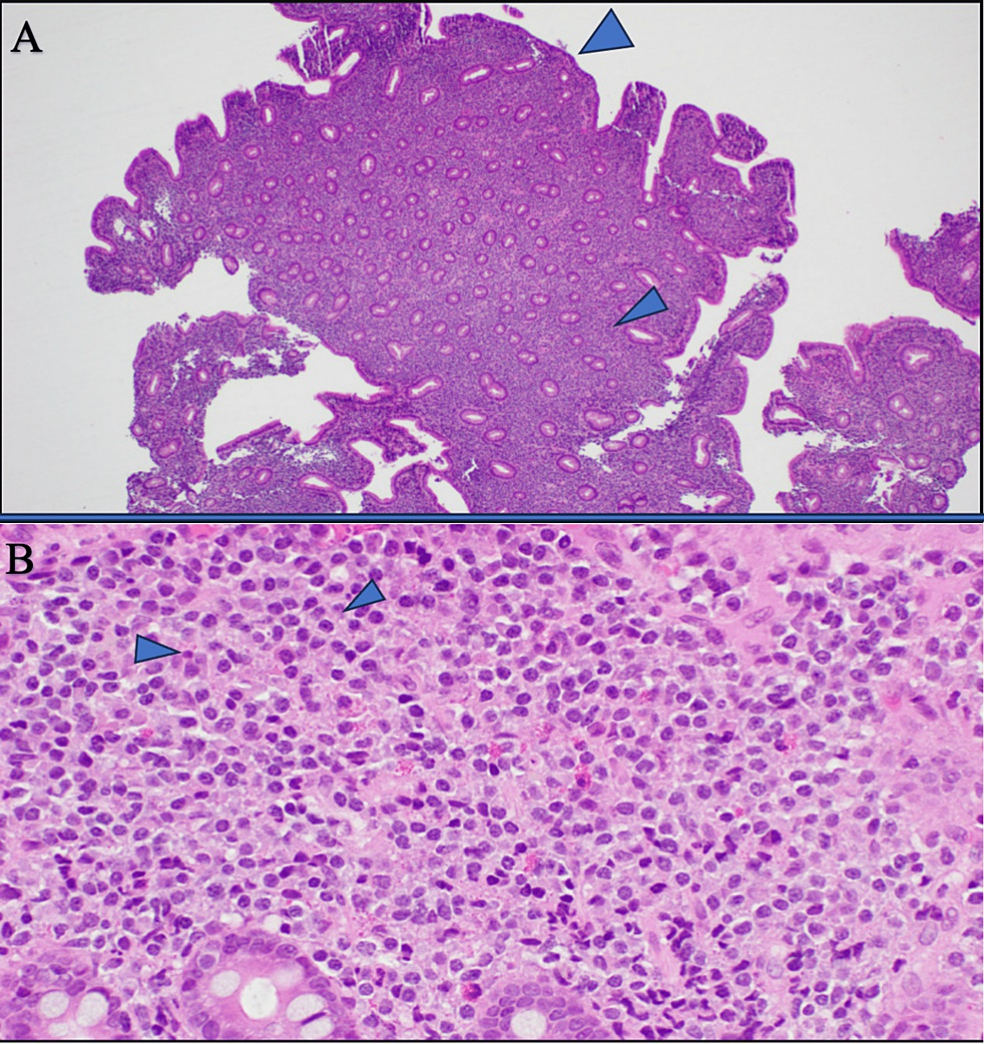
Microscopic histological patterns of IPSID (A) Small intestinal mucosa with marked villous blunting and expansion of lamina propria by small, mature lymphoid cells (H&E, low power magnification). (B) Small intestinal mucosa showing sheets of predominantly mature lymphoid cells with scattered plasma cells and eosinophils (H&E, high power magnification).

Capsule endoscopy revealed edema and hyperemia of the small bowel walls with intermittent areas of inflammation. Follow-up push enteroscopy with multiple small bowel biopsies showed diffuse lymphoplasmacytic infiltration of the mucosa with focal lymphoid clusters, blunting and focal flattening of villi, intraepithelial lymphocytosis, and mild crypt hyperplasia with plasma cell infiltration of the lamina propria (Figure 2A, 2B). This microscopic picture indicates a diagnosis of IPSID, ruling out lymphoma at this time. 

An octreotide scan highlighted intense uptake in the mesenteric lymph node previously noted in CT and gallium studies. Her symptoms continued to improve with levofloxacin and metronidazole, and a biopsy of the mesenteric lymph nodes revealed reactive lymphadenitis.

**Figure 3:**
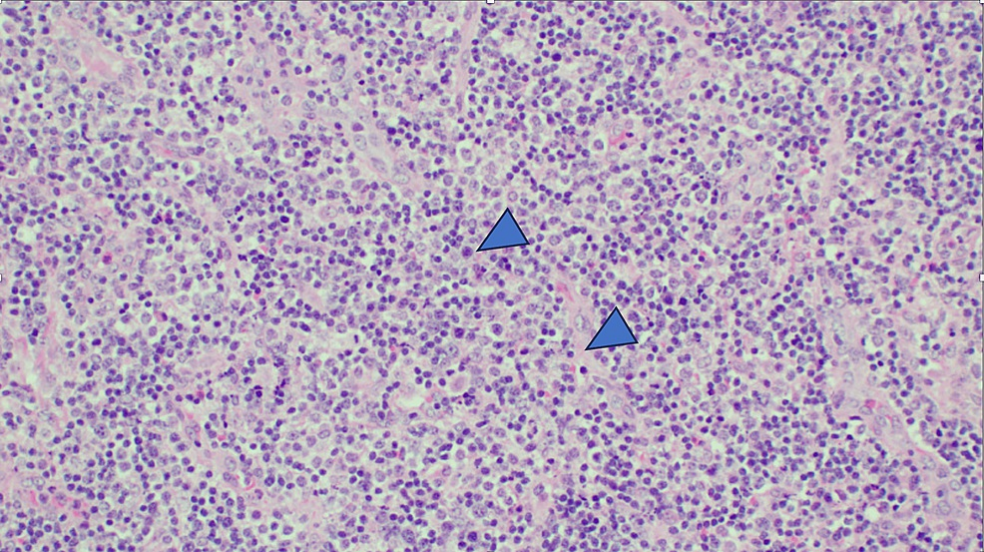
Microscopic histological patterns of lymphoma Cervical lymph node biopsy shows a polymorphous population of lymphocytes in a background of vascular proliferation (H&E, medium power magnification).

Approximately one year later, she presented with neck pain and difficulty swallowing, alongside her prior symptoms. Cervical and inguinal lymphadenopathy with increased uptake on PET-CT was found. A biopsy of the cervical lymph node revealed lymphoma, and she was diagnosed with IPSID stage C leading to lymphoma (Figure 3) and initiated on CHOP (cyclophosphamide, doxorubicin, vincristine, and prednisone) chemotherapy. Unfortunately, the patient succumbed to her disease shortly thereafter.

## Discussion

IPSID is a MALT lymphoma that develops in the small intestine; it is two to four times more common in males than in females [2] and most commonly presents in the third decade of life [5]. It is associated with poor sanitary conditions and is most often reported in the Mediterranean Basin, the Middle East, Africa, and Southeast Asia. Several studies have attempted to link IPSID with a bacterial cause, specifically repeated infections with *H. pylori *and *C. jejuni*. Early studies using culture-based methods failed to identify any pathogenic bacterial species linked to IPSID. Recently, the use of molecular techniques has provided further support for the link between *C. jejuni* and IPSID [3].

The pathogenesis of IPSID is thought to be similar in nature to MALT. It is hypothesized that in genetically and environmentally predisposed individuals, immunoparesis of the humoral and cytotoxic immune system leads to persistent *C. jejuni* infection resulting in chronic inflammation [3,6]. This leads to chronic antigenic stimulation, a proliferation of IgA α-heavy chain-producing plasma cells, and pervasive chromosomal damage, which eventually induces the development of intestinal lymphomas [1,3]. While this is the leading hypothesis, it has not been confirmed in animal models [6].

The prevalence of IPSID was estimated to be as high as 78% of all small intestinal malignancies as of the 1970s but has shown a recent decline that is likely related to improved sanitary conditions and decreased repeated exposure to gastrointestinal pathogens [7]. IPSID staging ranges from benign (stage A) to malignant (stage C) lesions. Benign lesions often show plasmocytic infiltrates with a marginal zone consisting of CD20+ cells. Stage B, often defined as an intermediate lesion, consists of villous atrophy, lymphoid aggregates, and large atypical immunoblastic cells. Finally, malignant lesions are large B-cell high-grade lymphomas with prominent plasmacytoid differentiation [4].

Early-stage IPSID (stages A and B) can often be completely treated with antibiotic therapy, while later stages (stage C) may progress to B-cell lymphoma, necessitating combination chemotherapy [8]. The exact point at which antibiotic treatment becomes ineffective is not well defined. In a review of 28 cases from 1991 to 2008, Pervez et al. reported initial responsiveness to antibiotic treatment across all stages, with follow-up biopsies showing complete or partial regression. However, three cases later developed into diffuse large B-cell lymphoma, requiring chemotherapy. Thus, close monitoring is essential for all IPSID patients, even those in the early stages of receiving antibiotic therapy [4,9,10].

In the initial stages, distinguishing IPSID from other chronic inflammatory processes that cause mesenteric lymphadenopathy can be challenging (Table 3). IPSID is often misdiagnosed as a celiac disease due to similar pathological findings, such as villous atrophy and lymphoplasmacytic infiltrates. In our case, a final diagnosis of stage C IPSID unresponsive to antibiotics was made based on clinical and histopathological findings, the patient's West African origin, and a negative celiac disease and infectious workup except for recurrent positive *Campylobacter* stool antigen. 

**Table 3:** Differential diagnosis of chronic diarrhea and mesenteric lymphadenopathy ^1^Rare manifestation of disseminated histoplasmosis typically seen in immunocompromised patients but has been reported in immunocompetent individuals with no obvious risk factors. ^2^Wide spectrum of infectious (~50%) and noninfectious (~50%) causes of diarrhea in HIV, including numerous opportunistic infections, antiretroviral therapy side effects, enteropathy from the destruction of CD4 T cells in gut-associated lymphoid tissue, malabsorption and pancreatic dysfunction from chronic illness, gastrointestinal lymphoma or Kaposi's sarcoma, and idiopathic HIV enteropathy. Etiologies may be distinguished in part by the chronicity and nature of gastrointestinal and associated extraintestinal symptoms, time since HIV infection, HIV viral load, and the most recent CD4 cell count. ^3^Syphilitic gastrointestinal involvement is rare and includes mucositis, gastritis, ileitis, colitis, proctitis, and hepatitis.

Disease	Symptoms	Mesenteric lymphadenopathy
Celiac disease	Chronic watery diarrhea associated with ingestion of gluten, constipation, abdominal pain/discomfort, weight loss, iron deficiency anemia, guaiac-positive stools, osteoporosis, dermatitis herpetiformis, neurological symptoms, hypoproteinemia, hypocalcemia, and elevated liver enzymes	Mainly in upper small bowel mesentery, follicular hyperplasia caused by autoimmune lymphocytic proliferation, low-attenuation, and cavitation in advanced symptomatic disease (rare)
Abdominal tuberculosis	Chronic abdominal pain/distension and diarrhea ± blood, malabsorption, constipation, nausea/vomiting, fever ± night sweats, weight loss, fatigue, abdominal mass, hepatomegaly, ascites, obstruction, intestinal hemorrhage, perforation, fistula, prior history of pulmonary tuberculosis (rare), abnormal chest radiographic findings (<50%)	Lymphadenopathy with central caseating necrosis (low-attenuation on CT), masses formed by conglomerating enlarged nodes, calcification in healed stage
Intestinal histoplasmosis^1^	Chronic watery diarrhea ± blood, constipation, tenesmus, abdominal pain, nausea/vomiting, fever ± night sweats, anorexia, weight loss, dysphagia, mucositis, hepatosplenomegaly, obstruction, intestinal hemorrhage, perforation, pancytopenia, hypoproteinemia, extraintestinal signs of histoplasmosis (e.g., cough, dyspnea, and varied skin lesions)	Macrophages containing intracellular yeast cells can be present in nodes throughout the mesentery.
Yersiniosis	Acute/subacute diarrhea ± blood, abdominal pain, nausea/vomiting, fever, pharyngitis, pseudoappendicitis due to terminal ileitis/mesenteric adenitis, leukocytosis, appendicitis, intussusception, perforation, gastrohepatic/splenic abscess, toxic megacolon, cholangitis	Mainly in the ileocecal region, lymphoid follicular hyperplasia with microabscesses
HIV^2^	Watery or bloody diarrhea ± abdominal pain that can be acute/subacute (HIV seroconversion) or chronic/acute-on-chronic (opportunistic infection, malignancy), other signs of acute HIV infection (eg, fever, weight loss, lymphadenopathy, rash), extraintestinal signs of AIDS-defining illnesses	More likely to be due to an opportunistic infection/malignancy than direct infection by HIV itself
Gastrointestinal syphilis^3^	Oral/anorectal lesions, dyspepsia, abdominal/anorectal pain, early satiety, vomiting, weight loss, pain on defecation, diarrhea, tenesmus, anal mucous discharge, intermittent rectal bleeding ± inguinal lymphadenopathy, extraintestinal signs of syphilis (eg, painless genital ulcer, maculopapular rash involving palms/soles)	Absent
IgG4-related disease	Subacute/relapsing-remitting course, mass/general enlargement of a single organ, lymphadenopathy, recurrent esophageal strictures, dysphagia, abdominal pain, diarrhea, weight loss, painless jaundice, new-onset diabetes, history of atopy, additional organ involvement (e.g. autoimmune hepatitis, retroperitoneal fibrosis, tubulointerstitial nephritis, interstitial pneumonia, salivary gland enlargement, orbital tumor, Hashimoto thyroiditis)	Several nodal histopathology patterns (eg, dense collagenous fibrosis and deposition of IgG4- positive plasma cells with lymphoplasmacytic infiltration)

Although our patient presented with common symptoms of IPSID including chronic diarrhea, weight loss, and epigastric pain, the diagnosis of IPSID was made after a protracted period of time. Initially, the etiology of our patient's symptoms was unclear, with a negative anti-TTG antibody assay, largely negative infectious workup, and improved mesenteric lymphadenopathy following antibiotics. The patient was discharged after antibiotic therapy and instructed to keep a food diary and follow up in the gastroenterology clinic. Unfortunately, her symptoms worsened post-discharge, leading to readmission. Prompt diagnosis and early treatment initiation are crucial, especially at stages responsive to antibiotics. IPSID should be considered in patients from endemic regions with classic symptoms, after ruling out other causes of diarrhea and mesenteric lymphadenopathy (Table 3).

In the case of our patient, *Campylobacter* stool antigen was persistently positive even after she had left an endemic region (West Africa) and received multiple courses of antibiotics, underscoring the puzzling nature of the source of her recurrent bacterial infection. Furthermore, her initial presentation and mesenteric lymph node biopsy and response to antibiotics suggested a diagnosis of IPSID stage B; however, she progressed to stage C with metastasis to the cervical lymph noses. Further studies are needed to understand the causes of progression in these patients and the cause of persistent stool antigens despite repeated prolonged antibiotic treatment.

## Conclusions

IPSID is a rare MALT lymphoma of the small intestine, more common in males and typically presenting in young adulthood. It is prevalent in regions with poor sanitation, such as the Mediterranean, Middle East, Africa, and Southeast Asia, and is linked to chronic infections, particularly *C. jejuni*. The importance of early diagnosis and timely treatment is crucial for improving patient outcomes. Future studies should focus on developing and refining diagnostic criteria and protocols to distinguish IPSID from other gastrointestinal disorders.

Our case highlights the persistence of *Campylobacter *infection despite multiple courses of antibiotics, underscoring the need to investigate the mechanisms behind antibiotic resistance in IPSID patients, which could lead to more effective treatment strategies. Understanding the factors that contribute to the progression of IPSID from early stages to high-grade lymphoma is essential, and research should explore genetic, immunological, and environmental factors that influence disease progression. The association between chronic infections, particularly with *C. jejuni*, and IPSID suggests a need for more detailed studies on the role of bacterial pathogens in the pathogenesis of this lymphoma.

Long-term studies on treatment outcomes, including the effectiveness of antibiotics and the need for chemotherapy in advanced stages, are necessary, and close monitoring protocols for early-stage patients should be developed to detect and manage progression promptly. Further epidemiological studies in endemic regions can provide insights into the prevalence, risk factors, and outcomes of IPSID, contributing to better public health strategies and resource allocation. These points underscore the need for a multifaceted research approach to enhance understanding, diagnosis, and treatment of IPSID, ultimately improving patient care and outcomes.
